# Adults have rather stable, yet domain-specific tendencies in social and temporal comparisons

**DOI:** 10.1038/s41598-026-70006-6

**Published:** 2026-09-05

**Authors:** Cornelia Wrzus, Gabriela Küchler, Kira S. A. Borgdorf, Corina Aguilar-Raab

**Affiliations:** 1 Psychological Institute, Heidelberg University, Heidelberg, Germany; 2 Network Aging Research, Heidelberg University, Heidelberg, Germany; 3 Institute for Medical Psychology of the Center for Psychosocial Medicine, Heidelberg University Hospital, Heidelberg, Germany; 4 Department of Clinical Psychology, Interaction- and Psychotherapy Research, Institute for Compassionate Awareness and Interdependence Research and Practice IN‐CARE, University of Mannheim, Mannheim, Germany

**Keywords:** Social comparison, Temporal comparison, Domain specificity, Adult lifespan, Self-evaluation, Neuroscience, Psychology, Psychology

## Abstract

Past research on how people evaluate and compare themselves in central characteristics focuses strongly on preconditions, processes, and consequences of such comparisons while neglecting individual differences. The current study bridges social and personality research by examining domain specificity, temporal stability, and age differences of social and past-temporal comparisons. Using lifespan samples from Germany (*N* = 313, 18–84 years, 49.8% female) and the USA (*N* = 302, 18–72 years, 52.0% female), participants reported the frequency and perceived usefulness of social and past-temporal comparisons concerning five domains: emotional stability, extraversion, physical appearance, professional success, and personal life. The results show that individual differences in comparison tendencies were rather domain-specific. Within a specific life domain, the usage of social and past-temporal comparisons was strongly correlated. Individual differences in comparison tendencies were moderately stable over four weeks (~ 0.60). With older age, participants indicated using comparisons less frequently and seeing them as less useful than young adults. We discuss that future research on social and temporal comparisons would benefit from a trait-state approach taking individual differences in domain-specific comparisons and the co-occurrence of social and temporal comparisons into account.

**Supplementary Information:**

The online version contains supplementary material available at https://doi.org/10.1038/s41598-026-70006-6.

## Introduction

Many people often compare themselves—with others, with a past or ideal self, or with other strengths they possess^1^. Much research has been devoted to understanding the preconditions and consequences of such social, temporal, or dimensional comparisons^1–3^. Also, the majority of previous studies focused on either social or temporal comparisons separately often in one specific domain. Thus, the focus on (a) situational influences of comparisons and (b) consequences of experimentally induced comparisons in selected domains has largely neglected an important question: To what extent do individuals possess general comparison tendencies that they apply consistently in different domains, such as concerning personality characteristics, physical appearance, or professional success? Relatively stable individual differences in comparison tendencies could alter situational influences as well as emotional and behavioral consequences of comparisons. For example, people who rarely engage in past-temporal comparisons might be less susceptible to feedback and change. Also, it is largely open, whether comparing oneself to others regarding one aspect, e.g., appearance, increases the likelihood to compare oneself in other aspects, e.g., job success, personality characteristics. Moreover, people may find comparing themselves to others useful and motivating concerning one domain, such as job success, whereas they might find it less useful or detrimental to compare to others or themselves in another domain, like their appearance^2,4,5^.

We thus extend previous research on individual differences in domain-unspecific social comparisons^6^ by addressing the domain specificity and temporal stability of both social and past-temporal comparisons in five previously studied major domains that broadly cover important life aspects^7^: physical appearance, professional success, and personal life, and two personality characteristics, specifically emotional stability and extraversion. We focus on social and past-temporal comparisons, excluding dimensional comparisons, because unlike in studies on (academic) performance, which often compare language and math skills, suitable domains for comparing physical appearance, personal, or professional success are difficult to apply. To generalize findings beyond young adulthood, which previous research on comparisons mostly focused on, we include lifespan samples from two countries and address age differences in social and past-temporal comparisons in these five domains.

### Social and past-temporal comparisons as personality differences

Comparisons are ubiquitous when individuals form self-perceptions of their characteristics and abilities (for reviews, see^1,2^) as comparisons are defined as “an examination of two or more variables to establish similarities and/or dissimilarities”^2^, p. 1285. Since the establishment of Festinger’s theory of social comparison processes^8^ and Albert’s theory of temporal comparisons^9^, much research has been devoted to the underlying motives of comparisons (e.g., self-enhancement, self-verification^10^, as well as affective, cognitive, and behavioral outcomes of comparisons (for overviews, see^1,11–13^). This research generally focused on social comparisons (i.e., with others, such as “I am more successful than my neighbor”) *or* temporal comparisons (i.e., with oneself in the past or future, such as “I am more successful than last five years ago”) *or* dimensional comparisons (i.e., with oneself on different characteristics, such as “I am not good with numbers, but with languages”). The role of comparison processes for individuals’ self-concepts of their characteristics has been heavily studied in the last decades, for example, concerning momentarily activated self-evaluation motives, (dis-)similarity with the comparison target, and other situational factors that elicit specific comparisons and consequences^1,12,13^.

Following general state-trait frameworks of human experiences and behavior^14–16^, it seems possible, yet not substantially studied, that individuals possess a general tendency to apply different kinds of comparisons more or less often. Similar to other individual differences that possess trait and state aspects such as Big Five traits, self-esteem, or well-being, people could differ consistently from each other in general comparison tendencies irrespective of certain life domains (i.e., trait aspects) and manifest specific comparisons in specific situations and regarding specific domains or content. Thus, such comparison tendencies would demonstrate both relatively stable individual differences between people, which contribute to momentary occurrences of comparisons (i.e., states) in specific situations. Past research on individual differences focused on the tendency for social comparisons^6,17^, and followed mainly a domain-unspecific approach (i.e., asking about the frequency of social comparisons in general). The past studies demonstrated temporal stability that was comparable to that of personality characteristics with retest correlations of *r* =.70 over four weeks and *r* =.60 over 12 months^4^, with somewhat lower values for specific comparisons concerning appearance and well-being^18^. To our knowledge, no study has quantified the retest stability of temporal comparisons, yet one could also expect similar stability as observed for social comparisons due to their overall functional similarity^9^.

In addition to some consistency over time, associations with established personality characteristics have been examined and demonstrated most convincingly for neuroticism and self-esteem^3,6,17^. Still, associations between a general social comparison orientation and personality characteristics leave open whether social comparisons are consistent across domains: whether someone who eagerly compares job successes would be prone to compare physical appearance and other personal characteristics to a similar extent.

Equally astonishingly, social and past-temporal comparisons have been examined rather separately^12,13^, presumably because they arose from separate theories^1,8,9^. Early on, it was speculated, but not empirically examined, that temporal and social comparisons work in concert and complement or substitute each other^9^. Similarly, in educational psychology, the interplay of social and dimensional comparisons has been explicitly addressed in the internal/external reference frame model for academic self-concept (e.g^19,20^). These lines of research (reviewed in Möller & Marsh, 2013) suggest that students often use different types of comparisons simultaneously (e.g., “I am better in math than last year, but worse than my fellow student”). Yet, how often and how much people use these comparisons usually depends on the domain, such as social skills or professional success. This is in line with findings that comparisons occur most often in areas that are personally important (e.g., social skills, professional success^21^).

In conclusion, the majority of previous studies has focused on either social or temporal comparisons, often in one or a few specific domains^3,4,18^. Thus, it remains open whether individuals apply one or both kinds of comparisons more frequently than others (i.e., as a trait-like characteristic), and specifically in different life domains or similarly across domains. Therefore, we examine the magnitude of the associations between social comparisons and past-temporal comparisons within and across previously studied domains: physical appearance, professional success, personal life, and personality^7,21^.

### Age differences in social and past-temporal comparisons

The vast majority of research on social or past-temporal comparisons focused on the evaluation of (academic) performance of students (i.e., adolescents or young adults^1,4^). Only a handful of studies examined the frequency of both social and temporal comparisons and consistently found that both younger and older adults report using past-temporal comparisons generally more frequently than social comparisons^7,21,22^. At the same time, some differences regarding the frequency of social and past-temporal comparisons occurred for distinct attributes (e.g., physical appearance vs. extraversion^21^). Specifically, regarding their aging or health, older adults used downward social comparisons more frequently than past-temporal comparisons^23–26^. Such favorable social comparisons might be especially relevant regarding the health and aging of older adults as most older adults experience declines in health over time^27,28^. This would lead to unfavorable past-temporal comparisons (i.e., being worse off compared to earlier times^29^).

Theoretical accounts of age differences in the use of comparisons agree that social comparisons should be more prevalent in adolescence and young adulthood due to the importance of peers and social standing among peers in this age period^30^. Later in adulthood, the reduction of social networks and the focus on close relationships^31,32^ could lead to fewer social comparisons^30^. The scarce empirical work supports these theoretical predictions and shows that young adults reported a stronger tendency to socially compare than older adults^6,22,33^, except concerning personal health^24^.

Theoretical accounts of age differences in past-temporal comparisons suggest that adolescents would use past-temporal comparisons less often than adults because (a) social comparisons are more relevant for adolescents, and (b) adolescents have a shorter time period to look back on^30^. This theoretical prediction received some empirical support^22,30^. Furthermore, it is plausible to assume that older adults use past-temporal comparisons less often than middle-aged adults because older adults focus more strongly on their remaining time instead of looking back^34^.

In summary, the limited research on age differences in social and past-temporal comparisons challenges earlier conclusions on the dominance of social comparisons that were derived from studies on young adults^35,36^. Lifespan theories regarding the changes in social relationships and time perspective suggest that with older age, both social and past-temporal comparisons become less important. Thus, further age-comparative research is needed to better understand the relevance of social and past-temporal comparisons across the entire adult lifespan and regarding multiple life domains.

### The present research

The present questionnaire-based study focused on studying the domain specificity, temporal stability, and age-related differences in both social and past-temporal comparisons in five domains: physical appearance, professional success, personal life, and two personality characteristics, that is, emotional stability and extraversion. We chose these domains because this research question was part of a larger study focusing on personality development and the role of comparison processes for the development in emotional stability and extraversion (*blinded*). The three additional domains covered important aspects of people’s lives, which have been addressed before^7,21^, and allowed for age comparisons.

Previous findings indicate that social and past-temporal comparisons represent distinct yet moderately related forms of self-evaluation^4,5^. Further, individuals rarely rely on a single reference standard, rather, social and temporal comparisons often complement each other^10^. At the same time, the tendency to engage in comparisons varies across life domains, occurring most frequently in areas that are personally important^14^ or which individuals desire to improve (*blinded*). Taken together, the simultaneous consideration of comparison tendencies by reference standard and life domain requests the specification of a bifactor model with two correlated general comparison tendency factors and five domain-specific factors reflecting differences in domain-specific comparisons beyond the two general comparison tendencies.

In addition to examining the frequency of social and past-temporal comparisons, we assessed the perceived usefulness of comparisons as a potential explanation for age differences in frequency: If individuals perceive comparisons as less useful, they might also use them less frequently. The perceived usefulness refers to participants’ subjective assessment whether the comparison has informational, motivational, or affective value. To generalize findings beyond a single country, we collected data from 615 participants between 18 and 84 years twice, four weeks apart in two countries, Germany and the USA, without specific assumptions on country effects regarding the main hypotheses. We preregistered the study, the following hypotheses, and the analyses (https://osf.io/amkc6/). The domain-unspecific social comparison orientation measure (INCOM^4^; was included later at the 4-week retest assessment as an additional criterion for validity for the newly developed items.

### Domain specificity, i.e., factorial structure

H1a: Comparisons are domain-specific.

H1b: Social and past-temporal comparison are separate, yet moderately positively correlated factors.

### Temporal stability

H2: Domain-specific social and past-temporal comparisons are as stable as other personality characteristics (i.e., characteristic adaptations, *r* >.60 over four weeks).

### Age differences

H3a: With older age, people use social comparisons less often, overall and in the specific domains.

H3b: With older age, people perceive social comparisons as less useful, overall and in the specific domains.

H3c: Age differences in the frequency of temporal comparisons follow an inverted u-shaped curve: Young adults and older adults use temporal comparisons less often than middle-aged adults, overall and in the specific domains.

H3d: Age differences in perceived usefulness of temporal comparisons follow an inverted u-shaped curve: Young adults and older adults perceive temporal comparisons as less useful than middle-aged adults, overall and in the specific domains.

## Results

Analyses were conducted with combined T1 data from both the U.S. and the German sample to examine the factor structure of comparisons (H1a, H1b) as well as age differences in comparisons (H3a-H3d). In addition, to estimate the retest stability of domain-specific comparisons, we analyzed retest correlations between T1 and the retest assessment four weeks later. On average, individuals in the USA reported using social and past-temporal comparisons more frequently in several domains (e.g., professional success, personality traits), and finding them more useful (OSM Table S4). Yet no substantial differences between countries occurred regarding the central hypotheses, except once when stated explicitly. Table 1 presents descriptive information on the central constructs. Associations between social and past-temporal comparisons are reported in the OSM Table S1.

**Table 1:** Descriptive Statistics and Retest Stability for Social and Past-Temporal Comparisons in Five Domains.

	Social comparisons			Past-temporal comparisons		
	M	SD	4-week retest *r*	M	SD	4-week retest *r*
Frequency						
Extraversion	3.67_a_	1.55	0.61 [0.55;0.67]	3.53 _b_	1.54	0.58 [0.52;0.64]
Emotional stability	3.28_a_	1.52	0.58 [0.51;0.64]	3.25_a_	1.59	0.55 [0.48;0.61]
Appearance	3.95_a_	1.48	0.70 [0.65;0.75]	4.13_b_	1.49	0.61 [0.55;0.67]
Professional success	4.30_a_	1.53	0.66 [0.60;0.71]	3.92_b_	1.57	0.61 [0.54;0.67]
Personal life	3.46_a_	1.58	0.64 [0.58;0.69]	3.71_b_	1.55	0.58 [0.52;0.64]
Usefulness						
Extraversion	2.91_a_	1.69	0.54 [0.47;0.60]	3.33_b_	1.82	0.54 [0.47;0.60]
Emotional stability	3.19_a_	1.79	0.48 [0.40;0.55]	3.41_b_	1.83	0.44 [0.36;0.52]
Appearance	2.82_a_	1.69	0.61 [0.55;0.67]	3.45_b_	1.82	0.56 [0.50;0.63]
Professional success	3.36_a_	1.70	0.57 [0.51;0.63]	3.59_b_	1.76	0.55 [0.48;0.61]
Personal life	2.63_a_	1.57	0.56 [0.49;0.62]	3.34_b_	1.80	0.48 [0.40;0.55]

Subscripts denote significant differences (*p* <.01) between social and past-temporal comparisons per row.

### Domain specificity of social and past-temporal comparisons

**Fig. 1:**
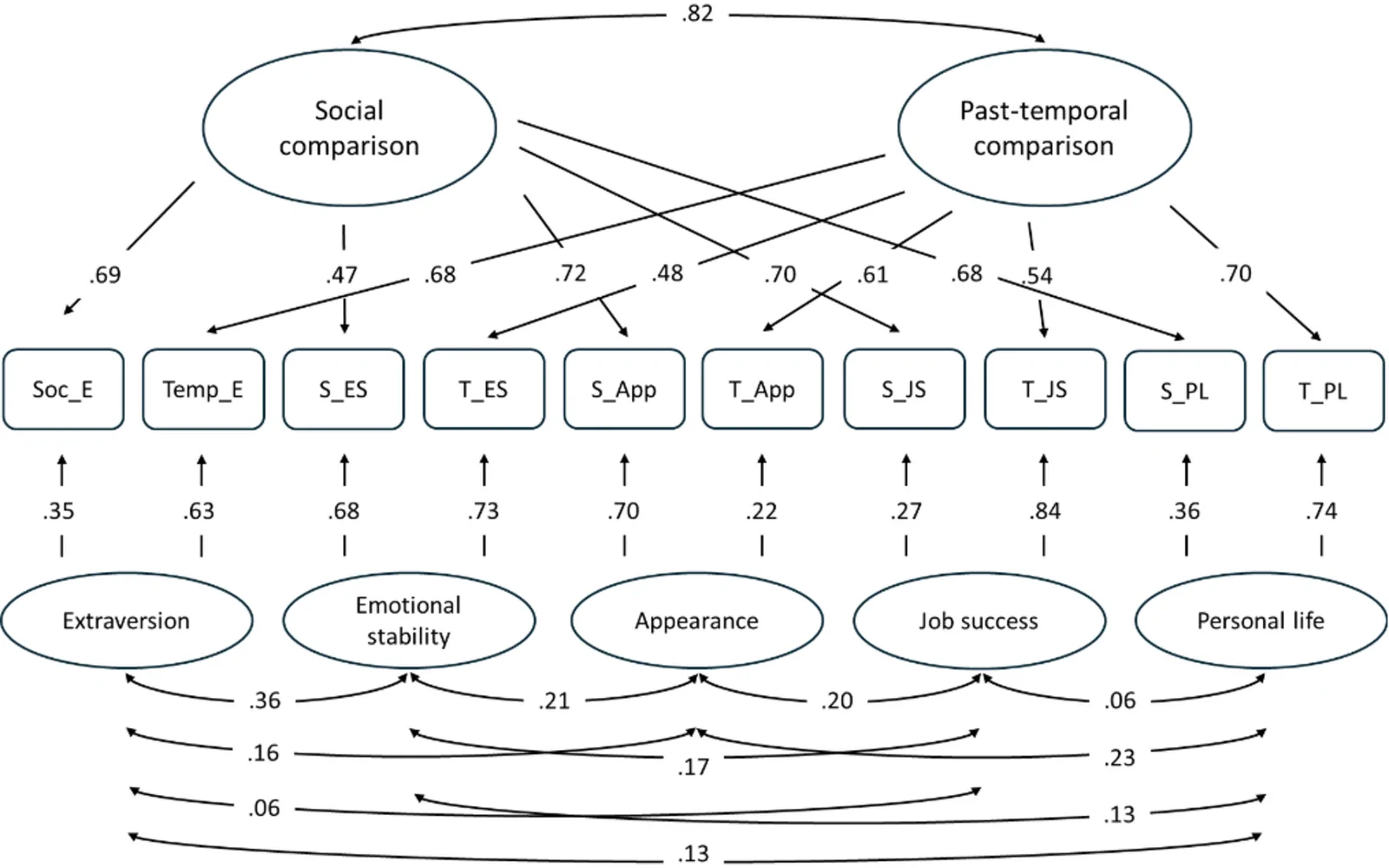
Bifactor Structural Equation Model of the Frequency of Social and Past-Temporal Comparisons in Five Domains. Factor indicators refer to the comparisons in five domains: Soc/S = social comparisons; Temp/T = past-temporal comparisons; E = extraversion; ES = emotional stability; App = physical appearance; JS = job success; PL = personal life.

To examine the structure of social and past-temporal comparisons in the five different domains, we specified separate bifactor models for the frequency and perceived usefulness of comparisons. The bifactor model^37^; Fig. 1) specified that answers regarding the frequency of certain comparisons reflect both a tendency to compare especially in certain domains (e.g., emotional stability, appearance) and a general tendency of using social or past-temporal comparisons irrespective of a certain domain. Accordingly, items load simultaneously on both a specific life-domain (i.e., domain-specific factor) and a specific kind of comparison (i.e., social or past-temporal comparison tendency). As a consequence, the life-domain specific factors are considered ‘residualized factors’^38^, and loadings can be interpreted as controlling for the general factors. Comparisons of this bifactor model with simpler models including only domain-specific or only reference-standard specific factors demonstrated the supremacy of the bifactor model (Table 2). Specifically, the bifactor model allowed the simultaneous test of hypotheses H1a and H1b. First, all items loaded substantially on the respective general factor for social or past-temporal comparisons (Fig. 1), which suggests that a large proportion of item variance is attributable to a common underlying construct. The domain-specific factors represent residualized dimensions, which capture variance in item responses beyond the two general factors. Some differences in loadings occurred in some domains (e.g., appearance, job success), which suggests that for these domains, the importance of the reference standards differs and much item variance is captured by the general factor.

**Table 2:** Model Comparisons for Alternative Models of Social and Past-Temporal Comparison Frequency in Five Domains.

Model	RMSEA	SRMR	CFI	Χ²	Δ Χ²
Frequency					
I Bifactor model	0.012 [0.00; 0.04]	0.015	0.999	18.46 (17), *p* = .36	-
II 2 factors for reference standards	0.180 [0.17; 0.19]	0.081	0.720	710.00 (34), *p* < .01	691.54 *p* <.01
III Higher-order factor and 2 factors for reference standards	0.180 [0.17; 0.19]	0.081	0.720	710.00 (34), *p* < .01	691.54 *p* <.01
IV 5 factors for domains	0.114 [0.10; 0.13]	0.042	0.918	223.75 (24), *p* <.01	205.29 *p* <.01
V Higher-order factor and 5 factors for domains	0.111 [0.10; 0.12]	0.048	0.906	258.10 (30), *p* <.01	239.64 *p* <.01
Usefulness					
I Bifactor model	0.051 [0.03; 0.07]	0.025	0.987	53.97 (21), *p* < .01	-
II 2 factors for reference standards	0.163 [0.15; 0.18]	0.070	0.776	592.28 (34), *p* < .01	538.31 *p* <.01
III Higher-order factor and 2 factors for reference standards	0.149 [0.14; 0.16]	0.079	0.791	510.63 (35), *p* < .01	456.66 *p* <.01
IV 5 factors for domains	0.110 [0.10; 0.12]	0.035	0.925	212.28 (25), *p* < .01	158.31 *p* <.01
V Higher-order factor and 5 factors for domains	0.108 [0.10; 0.12]	0.043	0.913	246.53 (30), *p* < .01	192.56 *p* <.01

Δ *Χ²* denotes the Chi-Square difference between the bifactor model and the model in the respective row. A significant difference implies that the compared models differ in their fit, giving preference to the better-fitting model (i.e., the bifactor model).

Results support H1a that the comparison tendencies are domain-specific because the correlations between the domain-specific factors were generally low (Fig. 1). Further supporting domain-specificity of comparisons, the models combining domain-specific indicators in only two reference-standard specific factors (i.e., social and past-temporal comparisons) fitted the data worse (Table 2, Models II & III). Similarly, models ignoring the kind of comparison (i.e., social or past-temporal comparison) and specifying only domain-specific factors also did not fit the observed data well (Table 2, Models IV & V).

Hypothesis H1b, which specified that social and past-temporal comparison are separate, yet moderately positively correlated factors, received only partial support. On the one hand, the bifactor model with two separate, yet correlated factors for social and past-temporal comparisons, fitted the data better than a model with only a higher-order factor combining both kinds of comparison (Table 2, Model III). Thus, social and past-temporal comparisons are statistically separable. On the other hand, the correlation between the two latent factors was substantially higher than expected, *r* =.82, 95% CI [0.77; 0.86], (Fig. 1).

The explained common variance (ECV) of the general factors was 0.53, ωH was 0.62, and the percent of uncontaminated correlations (PUC) was 0.89, indicating that substantial portions of the total item variance reflect the two general factors, while the domain-specific factors also capture noticeable portions of the item variance. This further supports the selected bifactor model because models that ignored either general comparison-standard factors or domain-specific factors fitted the data worse. These results imply that both aspects of comparisons are relevant and neither the domain nor the reference standard can be ignored.

In summary, how frequently individuals compare themselves with others or themselves in the past seems to be rather specific for a certain life domain (i.e., personality characteristics, physical appearance, professional success). Still, within each domain, individuals who use social comparisons more often also use past-temporal comparisons more often.

Regarding the factorial structure of the usefulness of comparisons, we observed similar results: the bifactor model fitted the data better than alternative models (Table 2, lower half). Due to estimation problems, the usefulness of comparisons regarding physical appearance could not be modeled as a separate factor, which we address in the discussion. Still, comparisons in this domain also served as indicators for the two general kinds of comparisons, that is, social and past-temporal comparisons. Similar to the SEM on the frequency of comparisons, the perceived usefulness of social and past-temporal comparisons was strongly correlated, *r* =.78, 95% CI [0.73; 0.84], indicating that the more individuals perceived social comparisons as useful, the more useful were past-temporal comparisons for them. This also applied to the perceived usefulness of specific comparison domains, with correlations around 0.30, except for a stronger association between the perceived usefulness of comparisons regarding extraversion and emotional stability, *r* =.58, 95% CI [0.49; 0.67] (OSM Figure S1).

### Retest stability and behavioral prediction of social and past-temporal comparisons

We assumed (H2) that social and past-temporal comparisons in the specific domains are as stable as other personality characteristics and thus demonstrate a 4-week retest stability of at least 0.60. Table 1 shows that this hypothesis was supported regarding the frequency of social and past-temporal comparisons in all five domains, as the 95% CIs included 0.60 in all cases. The 4-week stabilities of the perceived usefulness of comparisons were generally within a similar range with 95% CI also including 0.60, except for comparisons regarding emotional stability and past-temporal comparisons of one’s personal life, thus supporting H2 altogether with 85% of the retest-coefficients (Table 1). Also, the retest-stabilities of social comparisons were numerically higher compared to past-temporal comparisons for some domains, still, the confidence intervals overlapped, leaving numerical differences irrelevant.

Six months after T1, people were asked whether they wanted to know their prior personality scores (past-temporal comparison) or the personality scores of the other participants (social comparison), which served as a behavioral indicator of comparison tendencies. The majority of individuals (70.4%, *n* = 212) chose the past-temporal comparison, while the remaining participants (29.6%, *n* = 89) chose the social comparison. We conducted exploratory logistic regression analyses with the combined frequency and usefulness of comparisons regarding extraversion and emotional stability as predictors to determine whether individuals with a stronger social or past-temporal comparison tendency were more likely to view their own or others’ personality scores. The results indicated that individuals with a stronger tendency towards social comparisons were more likely to select others’ personality scores (OR = 1.65, *p* =.045). Additionally, participants from Germany were generally more likely to choose viewing others’ scores (country OR = 11.93, *p* =.02), yet nationality did not significantly moderate the effect of social comparison tendency (OR = 0.66, *p* =.10). Interestingly, U.S. participants with a stronger tendency towards past-temporal comparisons regarding personality traits more likely also chose to view others’ scores (OR = 1.71, *p* =.02), whereas no such association was observed among Germans (OR = 1.01, *p* =.93; interaction country-by-past temporal comparison OR = 0.59, *p* =.04). As country differences were not hypothesized and might be chance findings, further replication is needed before discussing potential country effects.

### Age differences in social and past-temporal comparisons

**Fig. 2:**
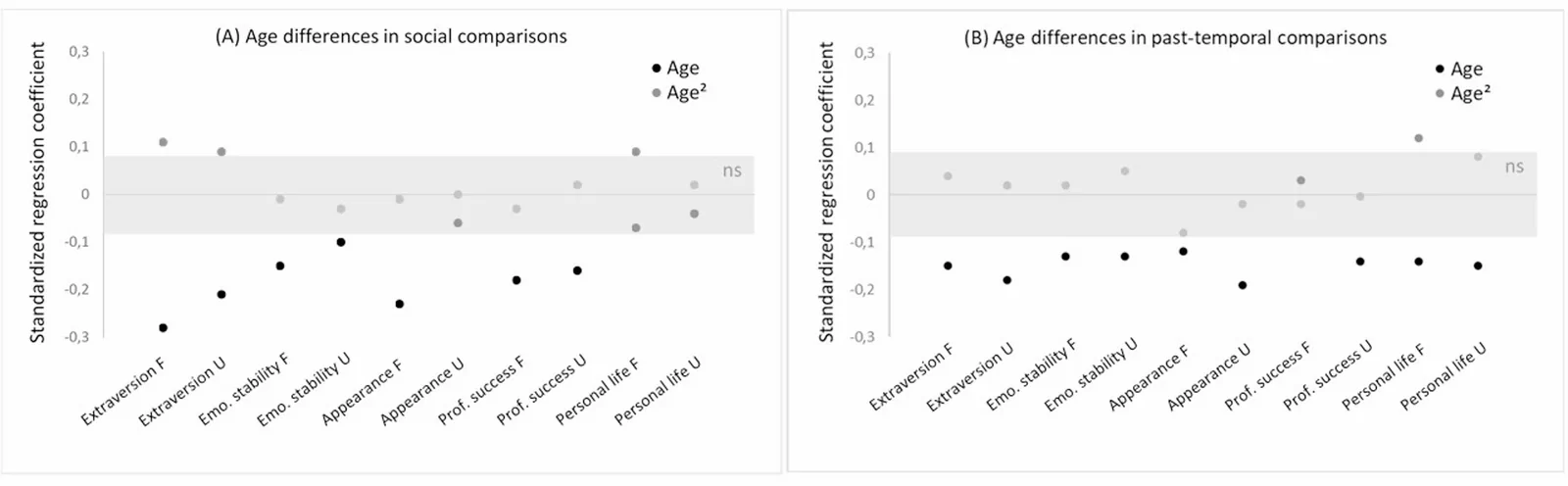
Age Differences in the Frequency and Usefulness of (**A**) Social Comparisons and (**B**) Past-Temporal Comparisons in Five Domains. F = frequency, U = usefulness.

To examine age differences in the frequency and usefulness of social and past-temporal comparisons, we conducted separate OLS regression analyses where age was used as linear and quadratic predictor and the comparison variables assessed at T1 were the outcomes (i.e., 5 domains by 2 reference standards, separate for frequency and usefulness). Confirming H3a and H3b, with higher age, individuals reported to use social comparisons less often (β = − 0.24, 95% CI [−0.32;−0.17], *p* <.01; *F*(1,594) = 37.7, *p* <.01; *R*² = 0.06) and perceived them as less useful (β = − 0.16, 95% CI [−0.24;−0.08], *p* <.01; *F*(1,594) = 14.72, *p* <.01; *R*² = 0.02), overall across the five domains. Adding age as a non-linear, quadratic predictor did not improve the models (frequency: *F*(1,593) = 1.06; *p* =.30; usefulness: *F*(1,593) = 0.38; *p* =.54). Results were similar when analyzing age differences separately for the five domains, except for non-significant age differences in the perceived usefulness of comparisons regarding appearance, personal life, and emotional stability (see Fig. 2A; full model results in OSM Table S2. Results of control analyses with all covariates are reported in OSM Table S4).

Regarding past-temporal comparisons across the five domains (H3c, H3d), with older age, individuals indicated to use past-temporal comparisons less often (β_age_ = − 0.15, 95% CI [−0.23;−0.07], *p* <.01; β_agesq_ = 0.04, 95% CI [−0.04;0.12], *p* =.33; *F*(2,593) = 6.62, *p* <.01; *R*² = 0.02), and to perceive them as less useful (β_age_ = − 0.21, 95% CI [−0.29;−0.13], *p* <.01; β_agesq_ = 0.04, 95% CI [−0.04;0.12], *p* =.32; *F*(2,593) = 12.8, *p* <.001; *R*² = 0.04). The same pattern was observed when analyzing age differences separately for the five domains (Fig. 2B, full model results in OSM Table S2 and Table S4 with covariates). Thus, the assumed non-linear age effect of past-temporal comparisons being less frequent (H3c) and less useful (H3d) for young and older adults compared to middle-aged adults was not supported because a linear age effect was observed: Past-temporal comparisons were used less frequently and perceived as less useful with older age.

### Explorative analysis

We explored how domain-specific social and past-temporal comparisons were associated with individual differences in social comparison orientation, as commonly assessed with the INCOM scale^6^. Other domain-specific social comparison scales were published^18^ after the study was conducted and thus not available during the data collection in fall 2021. Overall, with a higher general social comparison orientation, individuals compared themselves more frequently to others in the five specific life domains assessed in the current study (Table 3). As the INCOM scale covers diverse life domains to a different extent (e.g., performance, personal success), the associations were somewhat heterogeneous, but generally within a similar range from 0.51 to 0.61 (Table 3). In addition, the associations between the social comparison orientation and the frequency of domain-specific comparisons were numerically larger for social compared to past-temporal comparisons, although CIs often overlapped (Table 3). Since the INCOM scale largely focuses on the frequency of social comparisons (instead of perceived usefulness), the associations with perceived usefulness were smaller (Table 3).

In addition, we analyzed bivariate associations between the perceived usefulness of certain comparisons in a specific domain and the reported frequency in this domain (OSM Table S1). In general, the more social or past-temporal comparisons were seen as useful in a certain domain, the more often people engaged in them, with correlations ranging between 0.38 and 0.67 (*p*s < 0.01, Table S1). Since the direction of the association can be reciprocal, it is also plausible that when people engaged in certain comparisons more often, they then viewed such comparisons also as more useful.

**Table 3:** Bivariate Associations between Social Comparison Orientation and Domain-Specific Social and Past-Temporal Comparisons.

Social comparison	Social comparison orientation *r* [95% CI]	Past-temporal comparison	Social comparison orientation *r* [95% CI]
Frequency		Frequency	
Extraversion	0.61 [0.56;1.00]	Extraversion	0.46 [0.40;1.00]
Emotional stability	0.51 [0.44;1.00]	Emotional stability	0.33 [0.25;1.00]
Appearance	0.58 [0.53;1.00]	Appearance	0.45 [0.38;1.00]
Prof. success	0.57 [0.51;1.00]	Prof. success	0.38 [0.30;1.00]
Personal life	0.51 [0.44;1.00]	Personal life	0.42 [0.35;1.00]
Usefulness		Usefulness	
Extraversion	0.38 [0.31;1.00]	Extraversion	0.35 [0.27;1.00]
Emotional stability	0.42 [0.35;1.00]	Emotional stability	0.30 [0.23;1.00]
Appearance	0.34 [0.27;1.00]	Appearance	0.33 [0.26;1.00]
Prof. success	0.31 [0.24;1.00]	Prof. success	0.25 [0.17;1.00]
Personal life	0.33 [0.25;1.00]	Personal life	0.27 [0.19;1.00]

Social comparison orientation was assessed using the INCOM scale^6^; Confidence intervals (95% CI) are presented as asymmetric, one-sided CIs because positive correlations were assumed.

## Discussion

The current study aimed at bridging social and personality research in asking whether reliable individual differences exist in social and past-temporal comparisons, whereas other research often focused on momentary occurrences of comparisons, their antecedents, and consequences. Therefore, we examined the domain specificity vs. generality as well as temporal stability of both social and past-temporal comparisons concerning five relevant life domains. As we will discuss next, if some individuals engage in comparisons more frequently than others, irrespective of the domain of comparison, and if these individual differences are sufficiently stable over time, we could assume that individual differences in the tendency to compare can be considered as personality characteristics. Previous work using the domain-general INCOM scale^6^ demonstrated social comparison orientation as such a personality characteristic, similar to, for example, self-efficacy^3^. In these aspects, the current study extends previous research that focused strongly on the processes of comparisons, specifically antecedents and consequences, and single comparisons such as social or temporal comparisons in specific domains (e.g^1,2,4,7,9,36,39^).

The results of the current binational study offer three main conclusions: First, individual differences in comparisons are rather specific for a certain life domain, and within a domain, both social and past-temporal comparisons are similarly frequent, which largely confirms Hypotheses 1a and 1b. Specifically, the results support the domain-specificity of comparisons (H1a) because comparisons regarding five different domains could not be grouped into a single factor without decreasing the fit of the specified model relative to the observed data. That means, some individuals compare themselves more than others concerning their physical appearance, but not necessarily regarding their personality traits or professional success^7,21^. This aligns with recent findings on social comparisons regarding appearance and well-being^18^ as well as with research on envy, which rather occurs for a specific domain among individuals (e.g., appearance, wealth^40^).

Yet, within a domain, the frequency (and perceived usefulness) of both social and past-temporal comparisons were rather similar in the current study. Exceeding our assumption (H1b), social and past-temporal comparisons were strongly positively linked, thus people might use both social and temporal comparisons to similar extents despite referring to very different standards (i.e., other people or themselves), which is acknowledged in the distinct theoretical backgrounds^1,8,9^. Based on the current findings, one could conclude that individuals hardly have a “preferred mode of comparing” or a source of information (e.g., their earlier self), and use both kinds of comparisons similarly. This corresponds well with theoretical and empirical work from educational psychology on self-evaluations of academic performance, which concludes that different types of comparisons often co-occur^1^. For example, people might compare their success at work with how successful they were two years ago as well as how successful their colleague is.

The cooccurrence of social and temporal comparisons has potential implications for their research: Even when researchers manipulate or measure only one reference standard, people might engage in both kinds of comparisons in combination, and potentially offset the consequences of unfavorable comparisons. For example, while a disadvantageous social comparison on personal success might lead to decreases in satisfaction, the effect could be offset by comparing oneself to a few years ago (i.e., past-temporal comparison). Similarly, adults might engage in strategic social comparison (“I am still healthier than my neighbor”) to counteract disadvantageous temporal comparisons (“My health is worse than five years ago”).

The observed high association between using social and past-temporal comparisons might explain the current results regarding the weak prediction of later behavioral choice (in addition to methodological concerns, which we address in the limitations section). Likely, individuals would have preferred to receive both their own and others’ personality scores because individuals tend to take multiple sources of information into account simultaneously^1^. Therefore, experimental forced-choice paradigms^4^, including the current study, likely deviate from how individuals use comparisons in daily life^7,41^. Thus, the current findings add to process-oriented lines of research by demonstrating that different reference standards should be studied simultaneously and that substantial individual differences in comparisons exist depending on the domain in which individuals do or do not compare themselves. Accordingly, an isolated focus on social or temporal comparisons, generalizations across domains, or even a generalized assessment as in the INCOM scale^6^, might not capture the phenomena of comparisons well^18^.

Second, generally supporting Hypothesis 2, the individual differences in comparison tendencies were similarly stable over the course of four weeks like some other personality characteristics (e.g., self-esteem, well-being; but not as stable as Big Five personality traits) which also possess trait and state components^42–45^. Although longer retest intervals should be implemented in the future to compare the size of the retest stability to effect sizes for self-esteem and Big Five traits over months and years^42–45^, the current findings already support and extend the scarce studies on the stability of comparisons in academic performance^35^, of social comparison orientation^6,18^, or of envy as negative emotional response to unfavorable social comparisons^40,46^. As a caveat, neither the previous nor the current study controlled for objective life circumstances, and stable pleasant or unpleasant life circumstances could (partly) explain stability in comparisons. For example, being satisfied with one’s own appearance likely contributes to the frequency and perceived usefulness of comparisons regarding physical appearance. Thus, if the satisfaction with one’s appearance remains stable across four weeks, so will comparisons. The perceived usefulness of comparisons was numerically, but not substantially, less stable. Thus, social and past-temporal comparisons seem to possess both momentary aspects and aspects in which individuals differ systematically over at least four weeks, and future research should take both into account when studying antecedents, consequences, and processes of comparisons.

The third main conclusion concerns age effects. While the majority of previous research on comparisons focused on students and young adults, the current findings of small, but consistent age differences further extend previous knowledge on social and past-temporal comparisons. Specifically, with older age, individuals reported using both social and past-temporal comparisons less frequently, as well as perceiving them as less useful compared to young adults, especially concerning extraversion and appearance. These findings concur with research on age differences in social comparison orientation, which focuses on abilities and general social comparisons^33,47^. In addition, the current findings tie in with research on envious responses to unfavorable social comparisons: Lower levels of envy with older age^46^ might result from older adults using social comparisons less often. Innovatively, we assumed that younger and older adults would use past-temporal comparisons less often and find them less useful than middle-aged adults, yet we did not observe such a non-linear pattern in past-temporal comparisons. One possible explanation could be that self-evaluation motives might generally decline across the lifespan as individuals increasingly know themselves better and have a more established sense of themselves^48–50^. Yet, different life domains might differ in importance across the lifespan, and the health domain might be an exception in which older adults compare themselves more frequently than young adults with others or themselves^24^. Still, with the current cross-sectional data, we cannot identify individual developmental trajectories, draw conclusions on the developmental origins of age differences, or rule out generational differences as explanations. For example, younger generations have more access and seem more attuned to comparing themselves with others, including through social media^51–53^.

Despite examining comparisons in a fairly large and heterogeneous sample from two countries and providing novel insights on the domain specificity, temporal stability, and age differences in comparisons, we note some limitations of the current study. First, we focused on social and past-temporal comparisons because these are most frequently used in daily life^21^, despite other comparisons being possible, such as with one’s standing on other dimensions (i.e., characteristics) or with future or ideal selves^1,2^. Notably, future studies might want to include such comparisons, distinguish between upward, downward, or lateral comparisons, and take additional life domains into account. The distinction between upward and downward comparisons is most relevant for affective and motivational outcomes of comparisons, when being better off than others or oneself in the past can lead to satisfaction, and comparing worse usually leads to dissatisfaction, but can also enhance efforts^1–4,9^. As the current study focused on the occurrence of comparisons instead of affective consequences, collapsing across upward, downward, and lateral comparisons might be less relevant, yet future studies might want to examine specifically age differences in the direction of social and temporal comparisons, which might vary between life domains^23–26^. With respect to life domains, we focused on physical appearance, professional success, private life, and two Big Five traits, which are highly desired for self-evaluation and change^54,55^. Yet, we cannot conclude whether the current findings generalize to other domains, such as academic performance, social relationships (“Am I a good parent/partner/friend?”), or health^1,24,29,56^.

Second, in future studies, we would improve both the sample and the methodological approach. The online sample was prone to dropout, with only 68% of 615 participants continuing in the four-week retest assessment, which might have affected the robustness of the retest coefficients and the prediction of choosing social or past-temporal feedback. Still, retest coefficients were based on the sizable sample of 421 participants, and dropout was largely independent of the key study variables. Also, the current sample allows only cross-sectional age comparisons, which can also reflect generational differences in social or temporal comparisons. Longitudinal studies would allow to map the individual trajectories, although the currently covered age range from 18 to 84 years would be difficult to implement in longitudinal studies. In addition, a multimethod approach combining self-reported comparisons, underlying motives, and multiple behavioral indicators from different domains (e.g., dwelling on information of others’ job successes, private life, personality development, or appearance) would allow stronger conclusions on the stability and sources of comparison tendencies. Also, the current study used only one item per domain and reference standard, and future studies might consider multiple items to increase the reliability of measures^18^. This would also enhance the estimation of the retest stability because it allows separating the stability of the constructs from measurement error and unsystematic measurement error might have reduced the retest coefficients.

Furthermore, we specifically took a trait perspective on comparisons to complement the process-oriented research prevalent in this research field^36,57,58^. Combining both perspectives, for example, through ecologically momentary assessments in daily life (i.e., EMA), would allow examining both momentary processes and individual differences simultaneously, and could propel this research forward^41,59^. Also, EMA approaches would allow assessing the absolute frequency of (aware) comparisons (e.g., once or multiple times per day), whereas we were limited to relative frequency estimations like *seldom* and *very often*, because retrospective assessments of absolute frequencies of internal states are often distorted through recall biases^60^.

To conclude, the present research demonstrates that individuals possess more or less pronounced tendencies to compare themselves to others and their previous self, which are rather specific for certain domains (e.g., physical appearance, personality traits) and similarly stable as many personality characteristics. In contrast to process-oriented experimental research, social and past-temporal comparisons were closely related and likely co-occur within a domain, which indicates that comparison processes in daily life might be more complex and less isolated from each other than theorized so far. The cross-sectional age differences suggest a developmental pattern that is in line with increasing autonomy and self-reliance across the adult lifespan, yet awaits further longitudinal evidence.

## Methods

### Transparency and openness

We report the determination of the sample size, all data exclusions, all manipulations (none), and all measures in the study, and we follow JARS (Kazak, 2018). All data, analysis code, and research materials are available at [https://osf.io/b7hjn/]. Data were analyzed using Mplus 8.6^61^, R version 4.3.3 (R Core Team, 2020), and jamovi 2.3.28^62^. The design, hypotheses, and analysis were preregistered (https://osf.io/amkc6/).

After the preregistration, we included a behavioral indicator of comparisons at the last assessment, which occurred six months after the study start. Yet, we did not add hypotheses on predictive validity because parts of the data had already been collected.

### Participants and procedures

A priori power analyses suggested samples of *N* = 330 per country to allow SEM analyses and to account for longitudinal attrition (see power estimation concerning the entire project with different research questions and planned analyses: https://osf.io/b7hjn/). Participants were 327 German-speaking and 321 English-speaking adults, who we recruited using the Clickworker platform to achieve stratified, age- and gender-diverse samples from a variety of socioeconomic backgrounds. After the exclusion of non-compliant participants (see below), the final samples consisted of 313 German adults aged 18 to 84 years (*M*_age_ = 43.32, *SD*_age_ = 14.91, 49.8% female, 48.9% male, 1.3% non-binary/prefer not to say; 43.8% with a university degree) and 302 U.S. adults aged 18 to 72 years (*M*_age_ = 41.32, *SD*_age_ = 13.94, 52.0% female, 47.0% male, 1.0% non-binary/prefer not to say; 61.6% with a college/university degree). The majority reported being in a romantic relationship (Germany 56.2%; USA 61.6%). Income after taxes was assessed in both countries in categories using country currencies: up to 1,000; 1,000–2,000; 2,000–3,000; 3,000–5,000; 5,000–7,500; 7,500 − 10,000; more than 10,000. The average per-person income was just above 3,000€ and 3,000USD in 2021.

Registered adults on the Clickworker platform received an invitation to the study “Behavior and experiences in everyday life”, and information that they will be asked to answer different questionnaires on personal behaviors and experiences repeatedly. Data collection of the first assessment T1 took part in August 2021 in Germany and October 2021 in the USA. Four weeks after T1, comparisons were assessed again to examine retest stability. Six months after T1, a final questionnaire (T2) occurred to measure the behavioral indicator of comparisons as well as personality traits again for a separate research question on personality development (*blinded for review*). For all three assessment points, which lasted about 30 min (T1, T2) and 10 min (Retest), participants received compensation of €11.50 or $13.30, respectively. This study was performed in line with the principles of the Declaration of Helsinki and all participants gave informed consent before participation. The Ethics Committee of the Faculty of Behavioral and Cultural Studies at Heidelberg University approved the research project (#report blinded).

For attrition analyses, we compared participants taking part in the retest four weeks after T1 (*N* = 421) with participants who dropped out after T1 (*N* = 194). Participants, who did not participate in the retest did not differ from the remaining participants in most analyzed variables, except that discontinuing participants were somewhat younger (39.8 vs. 43.4 years), and reported a few comparisons to be less useful (e.g., professional success, emotional stability; complete results regarding attrition are reported in Table S5 in the OSM).

### Measurements

#### Social and past-temporal comparisons in different domains

For the current research questions, we focused on the measurement of social and past-temporal comparisons in five domains, which were assessed at T1 as well as at retest four weeks later. A complete list of the assessed constructs is available at [https://osf.io/b7hjn/].

We assessed the frequency and perceived usefulness of social and past-temporal comparisons concerning five domains: emotional stability, extraversion, physical appearance, professional success, and personal life. In addition, we assessed the perceived outcome of comparisons, which were, however not relevant for the current research questions and addressed in another manuscript on mindfulness (*blinded for review*). The root of the questions remained the same and we only exchanged the domain and the comparison (i.e., for social comparisons “other people”, for past-temporal comparisons “yourself in the past”). The frequency of social comparisons was assessed using the questions: “How often do you compare yourself with how sociable other people are?” (extraversion); “…how calm other people are?” (emotional stability); “…how successful other people are in their job” (professional success); “…how the private life of other people is?” (personal life), “…how other people look” (physical appearance) using a 7-point answering scale (never, very seldom, seldom, sometimes, often, very often, always). Complete original items also for assessing past-temporal comparisons are available in the OSM S1.

The perceived usefulness of social and past-temporal comparisons was assessed with one question for each reference standard and each domain with the same beginning and only exchanged domain, respectively: “How useful do you find comparisons with other people/with yourself in the past regarding [your sociability, your calmness, your appearance, your professional success, your private life]?”. All questions were answered on a 7-point scale (1 = *not at all useful*, 4 = *neither/nor*, 7 = *very useful*). Again, the complete original items are available in the OSM S1.

#### Behavioral indicator of comparison tendencies at T2

At the last assessment T2, six months after T1, we asked participants whether they would like to see the average personality scores of the other 300 + participants or their own scores from the T1 assessment six months ago. In both cases, they were informed that detailed information would be provided regarding the meaning of the Big Five traits. The participants selected either “I would like to see my own scores from 6 months ago.” (past-temporal comparison) or “I would like to see the average scores of all participants.” (social comparison), and saw the respective scores and explanations on the next page without the possibility of going back and changing their choice.

#### Covariates

As covariates, we focused on demographic information and thus included the country of residence (0 = Germany, 1 = USA), education (−1 = no college education, 1 = college education), income (−1 = up to 3,000€/USD, 1 = more than 3,000€/USD), and partnership status (−1 = without partnership, 1 = romantic relationship). Furthermore, the domain-unspecific social comparison orientation was assessed with the Iowa-Netherlands Comparison Orientation Measure (INCOM^6^;) at the 4-week retest assessment.

### Data analyses and exclusions

As recommended for online assessments and preregistered, we checked non-compliant responses using multiple established criteria (Meade & Craig, 2012). Data were excluded, when participants failed more than two attention check items, had an unlikely short completion time (faster than 50% of the estimated total response time), or created multiple suspicious response patterns (e.g., the same answer on more than 10 consecutive items; Yentes & Wilhelm, 2018). Of the initial 648 participants (327 in Germany, 321 in the USA), we excluded 33 individuals (14 German and 19 U.S. participants). As preregistered, we defined outliers as *M* ± 3*SD* and identified no outliers in the examined variables.

The descriptive and correlational analyses were conducted using R version 4.3.3 (R Core Team, 2020), and jamovi 2.3.28, which is an R-based open-source software^62^. The hypotheses were independent from each other, therefore no correction for multiple testing was necessary^63^. Only within the four hypotheses H3a-H3d regarding age differences, the tests for the overall age effect and age differences in specific domains were hierarchically nested and therefore we applied the BH adjustment procedure^63^.

To examine the structure of comparisons in different domains, we used T1 data and estimated bifactor SEMs in Mplus 8.6^61^ according to the specifications described in^37^ and displayed in Fig. 1. Specifically, in the bifactor model (Model I), the frequency (or perceived utility) of comparisons in each domain were specified to load simultaneously on (a) a domain-specific factor and (b) a general comparison factor representing either social or past-temporal comparison tendencies. This allows to test, whether answers on the specific comparison items reflect both a tendency to compare especially in certain domains (e.g., emotional stability, appearance) and a general tendency of using social or past-temporal comparisons irrespective of a certain domain. We compared this model to several alternative models (see Table 2). Model II included only two correlated factors for social and past-temporal comparison tendencies, without domain-specific factors. Model III extended this model by adding one higher-order factor to account for shared variance between social and past-temporal comparison tendencies. Model IV specified five correlated factors representing comparisons within each domain, without distinguishing between social and past-temporal comparisons. Model V further introduced one higher-order factor over the five domain-specific factors, with social and past-temporal comparison tendencies loading on the same domain factors (exact code available at [https://osf.io/b7hjn/].

## Supplementary Information

Below is the link to the electronic supplementary material.

Supplementary Material 1

## Data Availability

All data, analysis code, and research materials are available at [https://osf.io/b7hjn/].
