# Supplementary material for: Adults have rather stable, yet domain-specific tendencies in social and temporal comparisons
Source: Sci Rep. 2026 Sep 5;16:27819. doi: 10.1038/s41598-026-70006-6 (PMC13546287; doi:10.1038/s41598-026-70006-6)
Supplement: Supplementary file 1 [file 41598_2026_70006_MOESM1_ESM.docx]

**Supplementary material to
Adults have Rather Stable, yet Domain-Specific Tendencies in Social and Temporal Comparisons**

Overview of supplementary material

[S1: Complete Item List for Social and Past-temporal Comparisons in Different Domains	2](#_Toc226639639)

[Figure S1: Bi-factor Structural Equation Model of the Perceived Usefulness of Social and Past-Temporal Comparisons in Five Domains	4](#_Toc226639640)

[Supplementary Table S1	6](#_Toc226639641)

[Supplementary Table S2	7](#_Toc226639642)

[Supplementary Table S3	8](#_Toc226639643)

[Supplementary Table S4	10](#_Toc226639644)

# S1: Complete Item List for Social and Past-temporal Comparisons in Different Domains

| **English Items** | **German Items** |
| --- | --- |
| Instruction: We sometimes look for information to evaluate our current standing on characteristics, skills, abilities, and opinions by comparing ourselves with other people or what we were like in the past. In the following, we would like to know how you evaluate yourself and your situation compared with other people currently and what you were like in the past.    How often do you compare yourself… | Manchmal suchen wir nach Informationen, um zu beurteilen, wie wir hinsichtlich unserer Charaktereigenschaften, Fähigkeiten oder Meinungen abschneiden. Um sich ein aktuelles Bild von sich selbst und verschiedenen Lebensbereichen zu machen, vergleichen wir uns daher gelegentlich mit anderen Personen oder damit, wie wir früher einmal waren. Im Folgenden interessiert uns, wie Sie Ihre aktuelle Situation und Ihr aktuelles Selbst im Vergleich zu anderen und zu Ihrem vergangenen Selbst einschätzen.  Wie oft vergleichen Sie sich in Bezug auf … damit… |
| *Social comparisons frequency* | *Soziale Vergleiche Häufigkeit* |
| with how sociable other people are | wie kontaktfreudig andere Personen sind |
| with how calm other people are | wie gelassen andere Personen sind |
| with how other people look | wie andere Personen aussehen |
| with how successful other people are in their job | wie erfolgreich andere Personen im Beruf sind |
| with how the private life of other people is | wie das Privatleben anderer Personen ist |
| *Past-temporal comparisons frequency* | *Zeitliche Vergleiche Häufigkeit* |
| with how sociable you were in the past | wie kontaktfreudig Sie früher einmal waren |
| with how calm you were in the past | wie gelassen Sie früher einmal waren |
| with how you have looked in the past | wie Sie früher einmal aussahen |
| with how successful you were in your job in the past | wie erfolgreich Sie früher einmal im Beruf waren |
| with how your private life was in the past | wie Ihr Privatleben früher einmal war |
| *Frequency 7-point answering scale*: never, very seldom, seldom, sometimes, often, very often, always) | *Häufigkeit 7-stufige Antwortskala*: nie, sehr selten, selten, manchmal, häufig, sehr häufig, immer |
|  |  |
| How useful do you find comparisons… | Wie nützlich finden Sie Vergleiche bezüglich… |
| *Social comparisons usefulness* | *Soziale Vergleiche Nützlichkeit* |
| with other people regarding how sociable you are | Ihrer Kontaktfreude mit anderen Personen |
| with other people regarding how calm you are | Ihrer Gelassenheit mit anderen Personen |
| with other people regarding your appearance | Ihres Aussehens mit anderen Personen |
| with how successful other people are in their job | Ihres beruflichen Erfolgs mit anderen Personen |
| with other people regarding your private life | Ihres Privatlebens mit anderen Personen |
| *Past-temporal comparisons usefulness* | *Zeitliche Vergleiche Nützlichkeit* |
| with yourself in the past regarding how sociable you are | Ihrer Kontaktfreude mit sich selbst vor einigen Jahren |
| with yourself in the past regarding how calm you are | Ihrer Gelassenheit mit sich selbst vor einigen Jahren |
| with yourself in the past regarding your appearance | Ihres Aussehens mit sich selbst vor einigen Jahren |
| with how successful other people are in their job | Ihres beruflichen Erfolgs mit sich selbst vor einigen Jahren |
| with yourself in the past regarding your private life | Ihres Privatlebens mit sich selbst vor einigen Jahren |
| *Usefulness 7-point scale*: 1 = not at all useful, 4 = neither/nor, 7 = very useful | *Nützlichkeit* *7-stufige Antwortskala*: 1 =gar nicht nützlich, 3= teils/teils, 7 = sehr nützlich |

# Figure S1: Bi-factor Structural Equation Model of the Perceived Usefulness of Social and Past-Temporal Comparisons in Five Domains

*Note*. Factor indicators refer to the comparisons in five domains: Soc/S = social comparisons; Temp/T = past-temporal comparisons; E = extraversion; ES = emotional stability; App = physical appearance; JS = job success; PL = personal life

# Supplementary Table S1

*Associations between Frequency and Perceived Usefulness of Social and Past-Temporal Comparisons in Five Domains*

|  | Frequency | | | |
| --- | --- | --- | --- | --- |
|  | Social Comparisons | | Past-Temporal Comparisons | |
| Usefulness | *r* | 95% CI | *r* | 95% CI |
| Extraversion | .48 | [.42;.54] | .57 | [.52;.62] |
| Emotional stability | .61 | [.56;.66] | .67 | [.62;.71] |
| Appearance | .38 | [.31;.45] | .41 | [.34;.47] |
| Professional success | .41 | [.35;.48] | .49 | [.43;.55] |
| Personal life | .54 | [.48;.59] | .57 | [.51;.62] |

# Supplementary Table S2

*Age differences in Social and Past-Temporal Comparisons in Five Domains* *Based on OLS Regression with BH-adjustment for multiple testing*

|  | Social Comparisons | | | | | | | | | | | | | |  | Past-Temporal Comparisons | | | | | | | | | | | | | |
| --- | --- | --- | --- | --- | --- | --- | --- | --- | --- | --- | --- | --- | --- | --- | --- | --- | --- | --- | --- | --- | --- | --- | --- | --- | --- | --- | --- | --- | --- |
|  | Extraversion | | | Emotional stability | | | Appearance | | | Professional success | | | Personal life | |  | Extraversion | | | Emotional stability | | | Appearance | | | Professional success | | | Personal life | |
|  | β | *p* | β | | *p* | β | | *p* | β | | *p* | β | | *p* |  | β | *p* | β | | *p* | β | | *p* | β | | *p* | β | | *p* |
| *Frequency* |  |  |  | |  |  | |  |  | |  |  | |  |  |  |  |  | |  |  | |  |  | |  |  | |  |
| Age | -.28 | .001 | -.15 | | .001 | -.23 | | .001 | -.18 | | .001 | -.07 | | .10 |  | -.15 | .002 | -.13 | | .002 | -.12 | | .013 | .03 | | .48 | -.14 | | .002 |
| Age² | .11 | .05 | -.01 | | .86 | -.01 | | .86 | -.03 | | .78 | .09 | | .13 |  | .04 | .57 | .02 | | .73 | -.08 | | .15 | -.02 | | .73 | .12 | | .05 |
| *Usefulness* |  |  |  | |  |  | |  |  | |  |  | |  |  |  |  |  | |  |  | |  |  | |  |  | |  |
| Age | -.21 | .025 | -.10 | | .05 | -.06 | | .20 | -.16 | | .025 | -.04 | | .43 |  | -.18 | .001 | -.13 | | .001 | -.19 | | .001 | -.14 | | .001 | -.15 | | .001 |
| Age² | .09 | .15 | -.03 | | .85 | .00 | | .99 | .02 | | .99 | .02 | | .99 |  | .02 | .83 | .05 | | .55 | -.02 | | .83 | -.004 | | .93 | .08 | | .30 |

*Note*. all *p*-values are BH-adjusted for multiple testing within the family of hypotheses (Bogomolov et al., 2021), i.e., within H3a, H3b, H3c, and H3d because it was stated “… more/less often, overall and in the specific domains“ (see Hypotheses in main text under “*The present research*”).

# Supplementary Table S3

*Age differences in Social and Past-Temporal Comparisons in Five Domains* *estimated as Residualized Factors in Bifactor Models*

|  | Extraversion | | Emotional stability | | Appearance | | Professional success | | Personal life | | Model fit | | |
| --- | --- | --- | --- | --- | --- | --- | --- | --- | --- | --- | --- | --- | --- |
|  | β | *p* | β | *p* | β | *p* | β | *p* | β | *p* | RMSEA | SRMR | CFI |
| *Frequency* |  |  |  |  |  |  |  |  |  |  | .055 | .038 | .980 |
| Age | .33 | <.01 | .13 | <.01 | .22 | <.01 | -.09 | .06 | .12 | .02 |  |  |  |
| Age² | .20 | <.01 | .03 | .61 | .02 | .75 | .01 | .79 | .17 | <.01 |  |  |  |
| *Usefulness* |  |  |  |  |  |  |  |  |  |  |  |  |  |
| Age | .23 | <.01 | .09 | .06 | - | - | .12 | .05 | .08 | .14 | .054 | .036 | .977 |
| Age² | .10 | .06 | .03 | .55 | - | - | .02 | .73 | .09 | .07 |  |  |  |

*Note*. Age effects differ from age differences observed in OLS regression (Tables S2 and S4) because the domain-specific factors from the bifactor model do not distinguish between social and past-temporal comparisons, and part of the variance has been captured by the general factors (i.e., social and past-temporal comparisons irrespective of the domain). Therefore, the domain-specific factors are residualized factors after the variance of the general factor(s) has been taken into account (Eid et al., 2017).

# Supplementary Table S4

*Age differences in Social and Past-Temporal Comparisons in Five Domains* including Covariates

|  | Social Comparisons | | | | | | | | | | | | | | |  | Past-Temporal Comparisons | | | | | | | | | | | | | |
| --- | --- | --- | --- | --- | --- | --- | --- | --- | --- | --- | --- | --- | --- | --- | --- | --- | --- | --- | --- | --- | --- | --- | --- | --- | --- | --- | --- | --- | --- | --- |
|  | Extraversion | | | Emotional stability | | | | Appearance | | | Professional success | | | Personal life | |  | Extraversion | | | Emotional stability | | Appearance | | | | Professional success | | | Personal life | |
|  | β | *p* | β | | | *p* | β | | *p* | β | | *p* | β | | *p* |  | β | *p* | β | | *p* | | β | *p* | β | | *p* | β | | *p* |
| *Frequency* |  |  |  | |  | |  | |  |  | |  |  | |  |  |  |  |  | |  | |  |  |  | |  |  | |  |
| Age | -.28 | <.01 | -.15 | | <.01 | | -.23 | | <.01 | -.17 | | <.01 | -.07 | | .13 |  | -.15 | <.01 | -.13 | | <.01 | | -.12 | .01 | .04 | | .40 | -.14 | | <.01 |
| Age² | .12 | .01 | -.01 | | .85 | | -.01 | | .77 | -.02 | | .69 | .07 | | .11 |  | .05 | .22 | .02 | | .64 | | -.06 | .16 | -.003 | | .95 | .12 | | .01 |
| Country | .19 | .03 | .02 | | .84 | | -.05 | | .57 | .31 | | <.01 | -.17 | | .06 |  | .27 | <.01 | .02 | | .83 | | .24 | .01 | .37 | | <.01 | -.17 | | .05 |
| Education | .02 | .79 | .02 | | .79 | | .07 | | .46 | .09 | | .30 | .09 | | .31 |  | .01 | .93 | -.01 | | .92 | | .08 | .37 | .09 | | .30 | .03 | | .73 |
| Partnership | .06 | .52 | -.01 | | .94 | | .08 | | .36 | .12 | | .17 | -.05 | | .60 |  | -.01 | .95 | .01 | | .92 | | .16 | .08 | .11 | | .23 | .07 | | .43 |
| Income | -.08 | .44 | -.04 | | .75 | | -.18 | | .09 | -.24 | | .03 | -.13 | | .22 |  | .03 | .75 | .07 | | .50 | | -.18 | .10 | -.20 | | .07 | -.02 | | .87 |
| *Usefulness* |  |  |  | |  | |  | |  |  | |  |  | |  |  |  |  |  | |  | |  |  |  | |  |  | |  |
| Age | -.21 | <.01 | .10 | | .03 | | -.07 | | .13 | -.16 | | <.01 | -.04 | | .42 |  | -.18 | <.01 | -.14 | | <.01 | | -.20 | <.01 | -.14 | | <.01 | -.16 | | <.01 |
| Age² | .10 | .02 | .01 | | .76 | | -.00 | | .93 | .03 | | .53 | .02 | | .67 |  | .02 | .67 | .08 | | .10 | | -.01 | .83 | .02 | | .71 | .10 | | .03 |
| Country | .22 | .01 | .17 | | .06 | | -.18 | | .05 | .13 | | .13 | .03 | | .72 |  | .12 | .18 | .14 | | .12 | | .03 | .72 | .20 | | .03 | -.01 | | .89 |
| Education | -.05 | .59 | .01 | | .93 | | .01 | | .89 | .03 | | .76 | -.10 | | .25 |  | -.04 | .62 | .05 | | .55 | | .15 | .09 | .08 | | .40 | .05 | | .55 |
| Partnership | .06 | .54 | .08 | | .39 | | .00 | | .98 | -.05 | | .61 | .04 | | .69 |  | -.01 | .95 | .08 | | .35 | | .10 | .28 | .07 | | .42 | .17 | | .07 |
| Income | -.07 | .54 | .03 | | .77 | | .06 | | .59 | .05 | | .64 | .04 | | .75 |  | -.04 | .70 | .10 | | .34 | | -.07 | .52 | .03 | | .76 | -.01 | | .90 |

*Note*. country of residence (0 = Germany, 1 = USA), education (-1 = no college education, 1 = college education), income (-1 = up to 3,000€/USD, 1 = more than 3,000€/USD), and partnership status (-1 = without partnership, 1 = romantic relationship).
Income after taxes was assessed in both countries in categories: up to 1,000; 1,000-2,000; 2,000-3,000; 3,000-5,000; 5,000-7,500; 7,500-10,000; more than 10,000. We created the binary variable based on the average per-person income, which was just above 3,000€ and 3,000USD in 2021. In the German sample, 75.7% reported a per-person income below 3,000€, 11.8% above 3,000€, and 12.5% did not indicate their income. In the US sample, 60.3% reported a per-person income below 3,000USD, 27.8% above 3,000USD, and 11.9% did not indicate their income.

# Supplementary Table S5

*Attrition analyses: T1 Differences in Demographic Characteristics as well as Social and Past-Temporal Comparisons of Participants Continuing or Discontinuing the Study*

|  | Continuing Participants | | Discontinuing (dropouts) | | | Comparison of continuing and discontinuing participants | | |
| --- | --- | --- | --- | --- | --- | --- | --- | --- |
|  | *M* | *SD* | *M* | *SD* | | *t* | *p* | d |
| Age | 43.4 | 14.2 | 39.8 | 14.7 | | 2.85 | .005 | 0.25 |
| Gender | 0.48 | 0.50 | 0.51 | 0.50 | | -0.73 | .466 | -0.06 |
| Romantic relationship (y/n)^a^ | 0.12 | 0.99 | 0.31 | 0.95 | | -2.24 | .025 | -0.20 |
| College (y/n)^a^ | 0.03 | 1.00 | 0.10 | 1.00 | | -0.90 | .370 | -0.08 |
| *Social comparisons frequency (F) and utility (U)* | | | |  | |  |  |  |
| Extraversion_F | 3.62 | 1.57 | 3.78 | 1.51 | | -1.19 | .235 | -0.10 |
| Emot. stability_F | 3.26 | 1.51 | 3.33 | 1.53 | | -0.56 | .573 | -0.05 |
| Appearance_F | 3.90 | 1.45 | 4.08 | 1.53 | | -1.35 | .179 | -0.12 |
| Prof. success_F | 4.21 | 1.51 | 4.48 | 1.53 | | -2.01 | .045 | -0.17 |
| Personal life_F | 3.58 | 1.54 | 3.20 | 1.63 | | 2.78 | .006 | 0.24 |
| Extraversion_U | 2.79 | 1.65 | 3.20 | 1.77 | | -2.77 | .006 | -0.24 |
| Emot. stability_U | 3.06 | 1.75 | 3.51 | 1.84 | | -2.88 | .004 | -0.25 |
| Appearance_U | 2.79 | 1.66 | 2.89 | 1.77 | | -0.67 | .502 | -0.06 |
| Prof. success_U | 3.20 | 1.64 | 3.72 | 1.78 | | -3.50 | .001 | -0.31 |
| Personal life_U | 2.58 | 1.55 | 2.74 | 1.60 | | -1.23 | .219 | -0.11 |
| *Temporal comparisons frequency (F) and utility (U)* | | | | |  |  |  |  |
| Extraversion_F | 3.46 | 1.53 | 3.70 | 1.53 | | -1.85 | .065 | -0.16 |
| Emot. stability_F | 3.17 | 1.56 | 3.42 | 1.63 | | -1.84 | .066 | -0.16 |
| Appearance_F | 4.03 | 1.45 | 4.35 | 1.55 | | -2.48 | .013 | -0.22 |
| Prof. success_F | 3.86 | 1.55 | 4.05 | 1.62 | | -1.37 | .171 | -0.12 |
| Personal life_F | 3.77 | 1.51 | 3.58 | 1.62 | | 1.40 | .161 | 0.12 |
| Extraversion_U | 3.24 | 1.82 | 3.53 | 1.79 | | -1.86 | .063 | -0.16 |
| Emot. stability_U | 3.24 | 1.80 | 3.81 | 1.84 | | -3.57 | .001 | -0.31 |
| Appearance_U | 3.34 | 1.80 | 3.72 | 1.84 | | -2.33 | .020 | -0.21 |
| Prof. success_U | 3.49 | 1.73 | 3.83 | 1.78 | | -2.26 | .024 | -0.20 |
| Personal life_U | 3.25 | 1.77 | 3.55 | 1.84 | | -1.90 | .057 | -0.17 |

*Note*. Romantic relationship and college were coded no = -1, yes = 1 to be included in analyses as weighted control variables. F = frequency, U = usefulness
